# Early Predictive Value of Infectious Markers for Ventilator-associated Pneumonia after Stanford Type A Aortic Dissection Surgery

**DOI:** 10.31083/RCM26002

**Published:** 2025-02-17

**Authors:** Huibiao Deng, Xiaohong Wu, Bo Peng

**Affiliations:** ^1^Department of Critical Care Medicine, Shanghai General Hospital, Shanghai Jiaotong University School of Medicine, 201620 Shanghai, China; ^2^Department of Outpatient Medicine, Shanghai University of International Business and Economics, 201600 Shanghai, China; ^3^Department of Cardiothoracic Surgery, The Second Affiliated Hospital of Guangzhou University of Chinese Medicine, 510120 Guangzhou, Guangdong, China

**Keywords:** ventilator-associated pneumonia (VAP), Stanford type A aortic dissection, neutrophil/lymphocyte ratio (NLR), procalcitonin (PCT), C-reactive protein (CRP), sputum smear

## Abstract

**Background:**

This study investigates the early predictive value of infectious markers for ventilator-associated pneumonia (VAP) after Stanford type A aortic dissection surgery.

**Methods:**

A retrospective review of the medical records of all patients with Stanford type A aortic dissection admitted to Shanghai General Hospital from July 2020 to July 2023 who received mechanical ventilation after surgery was performed. Patients were divided into infection and non-infection groups according to the presence of VAP. The clinical data of the two groups were compared. The early predictive values of procalcitonin (PCT), C-reactive protein (CRP), the neutrophil/lymphocyte ratio (NLR) and sputum smears for VAP were evaluated by receiver operating characteristic (ROC) curve analysis.

**Results:**

A total of 139 patients with Stanford type A aortic dissection were included in this study. There were 35 cases of VAP infection, and the VAP incidence rate was 25.18%. The CRP, PCT, and NLR levels in the infection group were more significant than those in the non-infection group (*p* < 0.05). The percentage of positive sputum smears was 80.00% in the infected group and 77.88% in the non-infected group. The ROC curve analysis revealed that the areas under the curve (AUCs) of PCT, the NLR, CRP and sputum smear were 0.835, 0.763, 0.820 and 0.745, respectively, and the AUC for the combined diagnosis was 0.923. The pathogenic bacteria associated with VAP, after Stanford type A aortic dissection, was mainly *gram-negative bacteria*.

**Conclusions:**

The combined application of the NLR, CRP, PCT and sputum smear is helpful for the early diagnosis of VAP after Stanford type A aortic dissection surgery to help clinicians make decisions about treating VAP quickly.

## 1. Introduction 

**Fig. 1.**
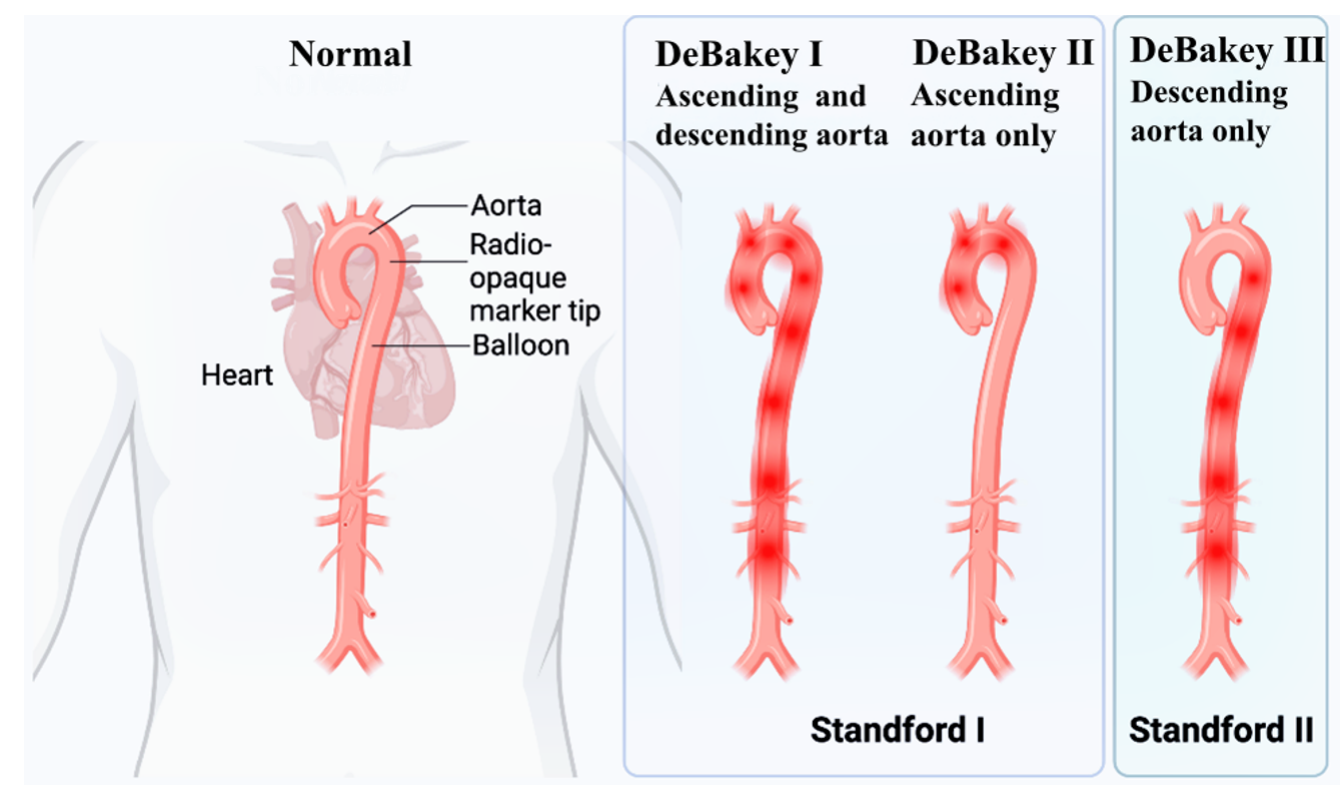
**Classification of acute aortic syndrome**. Stanford type A lesions involve the ascending aorta, whereas type B lesions are confined to the descending aorta. The DeBakey system accounts for pathology affecting both the ascending and descending aorta (I), only the ascending segment (II), or only the descending portion (III). [This figure was created with Eto icon (Lenovo version). ink].

Acute aortic dissection is an acute aortic disease caused by a tear in the inner layer of the aorta, forming a dissecting hematoma. It has the characteristics of acute onset and a high mortality rate. It affects multiple organ systems, including the heart, digestive tract and renal organs. Even if the disease is effectively controlled, patients typically require lifelong antihypertensive treatment to manage blood pressure and prevent recurrence. Aortic dissection can be divided into two main types: Stanford type A and Stanford type B, depending on the extent of aortic dissection involvement. Stanford type A dissections involve the ascending aorta, whereas Stanford type B dissections involve the thoracic descending aorta and its distal segments (Fig. 1) [1]. Acute type A aortic dissection (AAAD) is considered one of the most dangerous diseases in cardiovascular surgery, accounting for approximately 58–62% of aortic disease cases and almost 75% of acute aortic dissection cases. The mortality rate for patients without surgical treatment can reach 30% [2, 3].

Ventilator-associated pneumonia (VAP) is the most common nosocomial infection acquired in intensive care units (ICUs), with a mortality rate of up to 50%. Notably, at least 25% of these deaths are directly attributable to an infection rather than disease [4]. Surgical intervention is the only effective treatment for Stanford type A aortic dissection. However, such surgery can result in varying degrees of damage to the body’s circulation, airway, lung, diaphragm, chest wall, and intercostal muscles. Cardiac surgeries are often lengthy and highly invasive, increasing the incidence of postoperative infection, including VAP [5].

Therefore, early diagnosis and timely, appropriate interventions are crucial in clinical practice to reduce the risk of VAP and improve the postoperative prognosis of patients [6, 7]. Preventive strategies such as strict aseptic techniques during intubation, optimizing mechanical ventilation settings, and promoting early mobilization—can significantly mitigate these risks. Additionally, close monitoring for signs of infection and prompt antimicrobial therapy when necessary are essential components of postoperative care.

Identifying pathogens in patients with a pulmonary infection has always been a major challenge in medicine. Diagnostic methods such as sputum culture and next-generation sequencing are important diagnostic methods for pathogens associated with pneumonia; however, their clinical utility is often limited by long turnaround times for test results and high costs [8, 9]. Moreover, because of the serious condition of patients after Stanford type A aortic dissection surgery, radiological examinations are extremely inconvenient and carry additional risks.

Therefore, there is an urgent need in clinical practice to develop rapid and early diagnostic methods for VAP in this patient population. Early detection of VAP is crucial for timely intervention, reducing morbidity and mortality, and improving postoperative outcomes.

This study reports a retrospective case-control analysis of 139 patients who received mechanical ventilation after a standard operation for Stanford type A aortic dissection. The aim was to explore the application of infection markers in the early diagnosis of VAP and to investigate the distribution of pathogenic bacteria causing VAP in these patients. By evaluating the effectiveness of various infection markers and analyzing the bacterial profiles associated with VAP, this research seeks to provide valuable insights for clinicians.

## 2. Materials and Methods

### 2.1 Subjects 

**Fig. 2.**
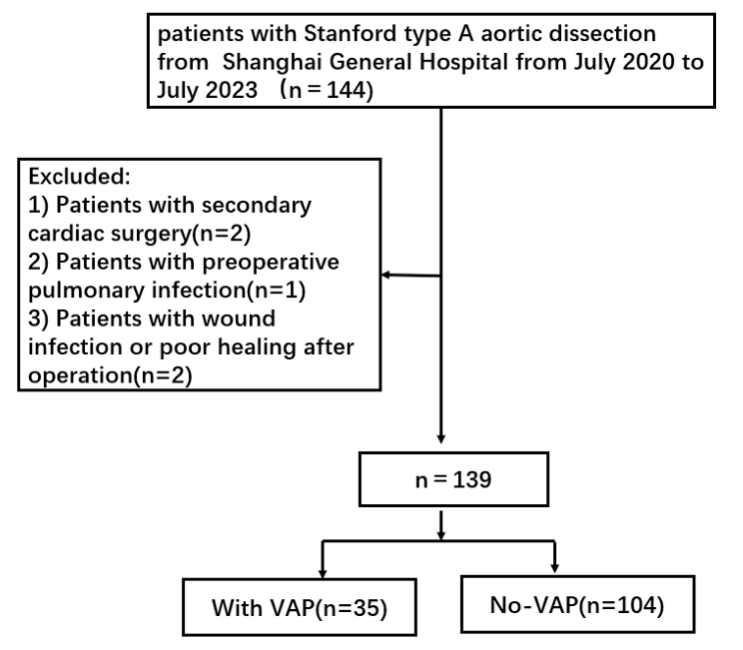
**The flow of patient selection**. VAP, ventilator-associated pneumonia.

The subjects for this study were selected from patients who received mechanical ventilation after undergoing surgery for Stanford type A aortic dissection at Shanghai General Hospital from July 2020 to July 2023. The inclusion criteria (Fig. 2) was as follows: (1) Patients who underwent a successfully completed type A aortic dissection; (2) Patients aged 18 years or older; (3) Patients who were receiving mechanical ventilation treatment; (4) Patients with complete clinical and follow-up data. The exclusion criteria were as follows: (1) Patients who underwent secondary cardiac surgery; (2) Patients in a critical condition before the emergency surgery; (3) Patients with preoperative pulmonary infection; (4) Patients who experienced wound infections or poor healing after the operation; and (5) patients with other major diseases or organ dysfunction.

Based on the presence of VAP after the operation, patients were divided into an infection group (n = 35) and a non-infection group (n = 104). The VAP diagnosis was based on the management guidelines of the European Respiratory Society (ERS)/European Society of Intensive Care Medicine (ESICM)/European Society of Clinical Microbiology and Infectious Diseases (ESCMID)/Asociación Latinoamericana del Tórax (ALAT) hospital-acquired pneumonia and ventilator-associated pneumonia [10]: (1) Development of pulmonary parenchymal infection within 48 hours after initiation of mechanical ventilation or within 48 hours after extubation. (2) A body temperature lower than 36 °C or higher than 38 °C. (3) A white blood cell count less than 4 × 10^9^/L or more than 10 × 10^9^/L; bronchial sputum culture that was positive. (4) A chest radiograph which revealed infiltrating lesions in the lung tissues. (5) Presence of abundant purulent secretions in the respiratory tract. This study was approved by the Ethics Committee of Shanghai General Hospital (Approval No. 2020125). All procedures adhered to the Declaration of Helsinki.

### 2.2 Data Collection 

Data were collected using the hospital’s electronic medical records management system. General patient information recorded included age, sex, body mass index (BMI), history of hypertension and diabetes, American Society of Anesthesiologists (ASA) classification, National Nosocomial Infection Surveillance (NNIS) risk index, type of surgery, perioperative antibiotic use, presence of implants, and whether extracorporeal membrane oxygenation (ECMO) support was required. Laboratory tests included blood counts, C-reactive protein (CRP), procalcitonin (PCT), low-density lipoprotein cholesterol (LDL-C), high-density lipoprotein cholesterol (HDL-C), creatine kinase-MB (CK-MB), brain natriuretic peptide (BNP), sputum smear, and sputum culture. All scores and test indices were based on the first postoperative measurements score and test index. Follow-up deadline for patients: (1) death; (2) cessation of mechanical ventilation within 48 hours.

Sputum smear and culture: Upon admission to the ICU after surgery, sputum samples were collected via bronchial aspiration for sputum smear microscopy and culture within two hours. Sputum culturing and smear microscopy were used to analyse the samples. Sputum smear tablet preparation was performed using a PREVITM Color Gram (Shanghai Hanfei Medical Equipment Co., Ltd., Shanghai, China) after Gram staining. To determine whether sputum samples were sufficient, samples containing fewer than 10 squamous cells and more than 25 polymorphonuclear leukocytes per low-power field were considered acceptable; insufficient samples were excluded from the study.

During microscopic examination, the number and distribution of bacteria were observed in detail. If more than four types of bacteria were present in the visual field, the sample was considered contaminated with pharyngeal flora. Bacteria were initially identified as pathogens if there were one to two dominant bacteria in the visual field, especially if these bacteria were clearly encapsulated, surrounded by, and accompanied by white blood cells, or if pus cells or neutrophils had phagocytosed the bacteria. Sputum smears were considered negative when no meaningful bacteria were found and positive when meaningful bacteria were detected. Sputum smears were prepared and interpreted by microbiological examiners with more than five years of experience.

For sputum culture, samples were sent to the laboratory within two hours of collection in a sterile container. A BD Phoenix100 automatic microbiological analyzer (Shanghai Jumu Medical Equipment Co., Ltd., Shanghai, China) was used for bacterial identification and drug sensitivity analysis. The Kirby–Bauer disk diffusion method was used to supplement and verify the drug sensitivity results. Drug sensitivity was determined according to the standards formulated by the American Clinical and Laboratory Standards Institute (CLSI) in 2020 [11]. The follow-up period for patients ended upon either the following: (1) death or (2) within 48 hours of the patient discontinuing mechanical ventilation.

### 2.3 Statistical Analysis

Data were summarized and organized using Excel 2010, and statistical analyses were performed using SPSS 22.0 software (IBM Corp., Armonk, NY, USA). Quantitative data are described as mean ± standard deviation ($\bar{x}$ ±  s), and an independent sample *t*-test was used to compare groups. Quantitative data with a non-normal distribution are expressed as M (Q1, Q3), and Wilcoxon rank sum test was used to compare differences between groups. Missing values for triglyceride (TG) were 2.9% and blood urea nitrogen (BUN) 3.6%. Missing data for these two continuous variables were handled via median imputation. There was no multicollinearity between the independent variables. Categorical data are presented as counts and/or percentages, and chi-square (χ^2^) tests were used for analysis. The receiver operating characteristic (ROC) curve [12] was used to evaluate the early predictive value of PCT, the NLR, CRP, and sputum smears for VAP after Stanford type A aortic dissection. The area under the curve (AUC) was compared with the Z score test, and *p* < 0.05 indicated that the difference was statistically significant.

## 3. Results

### 3.1 Univariate Analysis

A total of 139 patients who underwent surgery for Stanford type A aortic dissection were included in this study. Among these patients, 35 developed VAP, resulting in an incidence rate of 25.18%. The total duration of ventilator use across all patients was 1092 days, leading to a daily VAP infection rate of 31% per ventilator day.

There were no significant differences between the VAP group and the non-VAP group regarding sex distribution, age, BMI, requirement of ECMO support, history of diabetes or hypertension, ASA classification, type of surgery performed, NNIS risk index, presence of implants, or use of prophylactic antibiotics (*p* > 0.05) (Table 1).

**Table 1.** **Comparison of general characteristics between the two groups**.

Variable		VAP (n = 35)	No-VAP (n = 104)	t/χ^2^ value	*p*-value
Age (years)		52.18 ± 9.2	49.41 ± 10.90	1.350	0.179
Gender [n (%)]				1.061	0.303
	Male	25 (71.43)	83 (79.81)		
	Female	10 (28.57)	21 (20.19)		
BMI (kg/m^2^)		25.34 ± 4.4	25.28 ± 4.14	0.073	0.942
ECMO support [n (%)]				0.064	0.880
	Yes	2 (5.71)	3 (2.88)		
	No	33 (94.29)	101 (97.12)		
History of diabetes [n (%)]				3.225	0.073
	Yes	17 (48.57)	33 (31.73)		
	No	18 (51.43)	71 (68.27)		
Hypertension [n (%)]				2.482	0.115
	Yes	21 (60.00)	77 (74.04)		
	No	14 (40.00)	27 (25.96)		
Operation type [n (%)]				0.015	0.902
	Emergency treatment	30 (85.71)	90 (86.54)		
	Chosen date	5 (14.29)	14 (13.46)		
ASA score [n (%)]				0.021	0.884
	3–4	28 (80.00)	82 (78.85)		
	5	7 (20.00)	22 (21.15)		
NNIS score [n (%)]				0.120	0.729
	0–1	17 (48.57)	47 (45.19)		
	2	18 (51.43)	57 (54.81)		
Implants [n (%)]				0.060	0.807
	Yes	10 (28.57)	32 (30.77)		
	No	25 (71.43)	72 (69.23)		
Prophylactic medication [n (%)]				0.505	0.477
	Yes	2 (5.71)	10 (9.62)		
	No	33 (94.29)	94 (99.38)		

Note: BMI, body mass index; ECMO, extracorporeal membrane oxygenation; ASA, American Society of Anesthesiologists; NNIS, National Nosocomial Infection Surveillance Risk Index; VAP, ventilator-associated pneumonia.

**Fig. 3.**
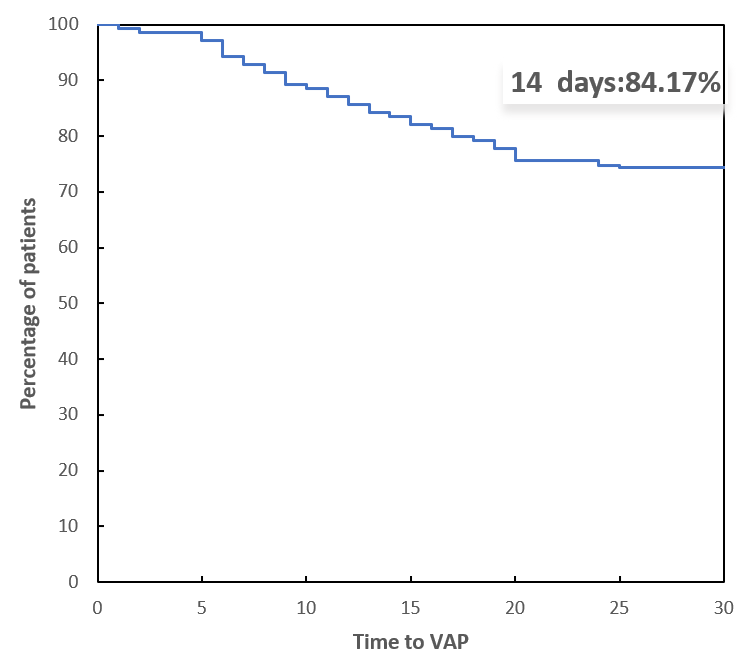
**Kaplan‒Meier curve showing the time of VAP after aortic dissection surgery**. VAP, ventilator-associated pneumonia.

According to the Kaplan–Meier curve presented in Fig. 3, the majority of VAP cases in patients who underwent surgery for Stanford type A aortic dissection occurred within two weeks after the initiation of mechanical ventilation. This finding suggests that the risk of developing VAP is highest during the early postoperative period, emphasizing the need for vigilant monitoring and preventive strategies during this critical time frame.

### 3.2 Comparison of Laboratory Test Indexes 

There were no significant differences between the two groups of patients who underwent surgery for Stanford type A aortic dissection regarding white blood cell (WBC) count, platelet (PLT) count, lymphocyte count, neutrophil count, LDL-C, HDL-C, BNP, or CK-MB levels. However, the levels of CRP, PCT, and the NLR were significantly higher in the infected group compared to the non-infected group (*p *  < 0.05). The percentage of positive sputum smears was 80.00% in the infected group and 77.88% in the non-infected group (Table 2).

**Table 2.** **Comparison of laboratory indicators between the two groups ($\bar{x}$ ± s)**.

Index		Infection group (n = 35)	Non infection group (n = 104)	*t/z*-value	*p*-value
WBC (×10^9^/L)		10.95 (5.30, 18.87)	10.00 (6.90, 14.80)	–0.431	0.066
PLT (×10^9^/L)		169.00 (135.75, 223.00)	192.00 (162.75, 237.25)	–1.602	0.109
Lymphocytes (×10^9^/L)		1.15 ± 0.37	1.22 ± 0.45	–0.840	0.401
Neutrophil (×10^9^/L)		8.24 ± 2.39	7.98 ± 2.35	0.560	0.576
NLR		9.66 ± 2.39	7.54 ± 1.95	4.189	<0.001
CRP (mg/L)		13.14 ± 3.22	8.65 ± 3.64	5.004	<0.001
PCT (ng/mL)		13.38 ± 3.03	8.83 ± 3.41	5.409	<0.001
LDL-C (mmol/L)		3.01 ± 0.42	3.08 ± 0.49	0.756	0.451
HDL-C (mmol/L)		1.08 ± 0.26	1.10 ± 0.29	0.362	0.718
CK-MB (U/L)		34.49 ± 6.95	32.51 ± 6.89	1.467	0.145
BNP (pg/mL)		120.29 ± 32.39	116.93 ± 34.31	0.510	0.610
TG (mmol/L)		0.97 (0.79, l.30)	l.04 (0.79, l.51)	0.127	0.902
TBIL (µmol/L)		10.19 (6.68, 12.85)	9.94 (6.80, 30.13)	0.401	0.689
ALB (g/L)		27.83 ± 14.11	27.05 ± 4.47	0.268	0.790
ALT (IU/L)		16.03 (8.95, 36.04)	16.51 (8.02, 30.97)	0.271	0.787
BUN (mmol/L)		1.89 (1.35, 2.95)	2.01 (1.43, 4.04)	0.361	0.718
CREA (µmol/L)		39.83 (31.95, 54.17)	43.25 (29.30, 58.24)	0.210	0.833
Sputum smear [n (%)]				37.774	<0.001
	Positive	28 (80.00)	23 (22.12)		
	Negative	7 (20.00)	81 (77.88)		

Note: WBC, white blood cell; NLR, neutrophil/lymphocyte ratio; PCT, procalcitonin; CRP, C-reactive protein; LDL-C, low-density lipoprotein cholesterol; HDL-C, high-density lipoprotein cholesterol; CK-MB, creatine kinase-MB; BNP, brain natriuretic peptide; TG, triglyceride; TBIL, total bilirubin; ALB, albumin; ALT, alanine transaminase; BUN, blood urea nitrogen; CREA, creatinine; PLT, platelet.

ROC curve analysis was performed to assess the early predictive value of PCT, the NLR and CRP results for VAP following surgery for Stanford type A aortic dissection. The AUCs of PCT, NLR, CRP, and sputum smear were 0.835, 0.763, 0.820, and 0.745 respectively. Significantly, when these four indicators, namely PCT, NLR, CRP, and sputum smear, were combined, the AUC rose to 0.923, suggesting a higher predictive value for the early diagnosis of VAP (Table 3).

**Table 3.** **Predictive efficacy of the NLR, CRP, PCT, and sputum smear for VAP after Stanford type A aortic dissection surgery**.

Index	AUC	Cutoff value	*p *value	95% CI	Sensitivity (%)	Specificity (%)
NLR	0.763	7.929	<0.001	0.643~0.879	73.70	80.40
PCT	0.835	9.719 (ng/mL)	<0.001	0.748~0.921	94.70	63.60
CRP	0.820	12.664 (mg/L)	<0.001	0.722~0.916	73.70	85.00
Sputum smear	0.745		<0.001	0.636~0.852	80.00	77.90
Union	0.923		<0.001	0.855~0.968	93.33	87.36

Note: NLR, neutrophil/lymphocyte ratio; PCT, procalcitonin; CRP, C-reactive protein; Union, NLR+PCT+CRP+sputum smear; AUC, area under the curve; VAP, ventilator-associated pneumonia.

### 3.3 Distribution of Pathogenic Bacteria

Among the 139 patients who underwent surgery for Stanford type A aortic dissection, 35 developed VAP, resulting in an incidence rate of 25.18%. In total, 80 strains of pathogenic microorganisms were detected in these patients. Of these, 71 strains were *gram-negative bacteria* (88.75%), 1 strain was a gram-positive bacterium (1.25%), and 8 strains were fungi (10%). The main pathogens identified were *Acinetobacter baumannii *(29 strains), *Klebsiella pneumoniae* (22 strains), and *Stenotrophomonas maltophilia* (9 strains) (Table 4).

**Table 4.** **Pathogenic bacterial distribution in VAP patients after Stanford type A aortic dissection surgery**.

Pathogenic bacteria	Number of plants (n = 80)	Composition ratio (%)
*Gram-negative bacteria*	71	88.75
*Acinetobacter baumannii*	29	36.25
*Klebsiella pneumoniae*	22	27.50
*Oligomonas maltophilia*	9	11.25
*Pseudomonas aeruginosa*	8	10.00
*Escherichia coli*	1	1.25
*Enterobacter cloacae*	2	2.50
*Gram-positive bacteria*	1	1.25
*Staphylococcus aureus*	1	1.25
*Fungus*	8	10.00

## 4. Discussion

Acute type A aortic dissection is a highly fatal cardiovascular emergency. The risk of postoperative infection is very high [13], leading to increased mortality, wastage of medical resources, an increased economic burden, and numerous other negative effects. Compared with bloodstream infections, urinary tract, surgical incisions, and other sites, pulmonary infection is the most common nosocomial infection in patients with acute type A aortic dissection, with most patients developing VAP [14]. In this study, a total of 139 patients with acute type A aortic dissection who required mechanical ventilation were included, among whom 35 developed postoperative VAP. The infection rate was 25.18%. Wang *et al*. [15] found that postoperative pneumonia developed in 170 of 492 patients (34.6%). The higher infection rate in their study compared to ours may be related to differences in patient selection and sample size, but both studies indicate a high incidence of VAP after surgery for Stanford type A aortic dissection.

Additionally, our study was conducted during the COVID-19 pandemic in China, whereas the study by Wang *et al*. [15] was conducted before the outbreak (between January 2016 and December 2019). Our hospital was not designated to treat COVID-19 patients during the pandemic; therefore, we did not encounter patients with aortic dissection complicated by COVID-19. Moreover, during the pandemic, unprecedented measures were implemented to prevent and control nosocomial infections, which may have contributed to the lower incidence of VAP observed in our study.

The NLR is an easily applicable, simple, rapid, and cost-effective marker calculated by dividing the number of neutrophils (cells/mL) by the number of lymphocytes (cells/mL). The NLR in peripheral blood reflects the immune status and degree of inflammation in the body [16, 17]. Qian *et al*. [18] reported that the negative predictive value of the NLR could reliably exclude postoperative infection, but conclusive evidence for predicting postoperative infection was insufficient. In our study, the NLR in the infected group was significantly higher than that in the uninfected group, with an AUC of 0.763, a sensitivity of 73.70%, and a specificity of 80.40%. These findings suggest that the NLR has predictive value for the occurrence of VAP after Stanford type A aortic dissection surgery, although its sensitivity is moderate.

CRP is an acute-phase response protein commonly used for the clinical evaluation and diagnosis of bacterial infection. It is synthesized and secreted by the liver in significant amounts under infection, tissue damage, and stress conditions. Subsequently, it can activate monocytes, lymphocytes, and macrophages, enhance the inflammatory response, and its levels rapidly increase within 24 hours of bacterial infection [19, 20, 21]. Thus, it has become a common indicator for diagnosing pathogenic infections. In this study, the CRP levels in the infected group were significantly higher than those in the non-infected group. The AUC for CRP in predicting VAP was 0.820, with a sensitivity of 73.70% and a specificity of 85.00%. Wussler *et al*. [22] reported an AUC for CRP of 0.82 (95% CI, 0.79–0.85), which is consistent with our findings.

PCT is a peptide precursor of the hormone calcitonin and serves as a biomarker for bacterial infections. It is a glycoprotein with no hormone activity. Under normal conditions, PCT is mainly produced by thyroid C cells, and its levels in the blood are low [23]. However, during systemic bacterial infections, various tissues and cells—including lymphocytes, monocytes, and macrophages—synthesize large amounts of PCT in a short period, leading to a sharp increase in blood PCT levels [24, 25, 26]. In this study, the AUC of PCT for predicting VAP was 0.835, with a sensitivity of 94.70% and a specificity of 63.60%. Luyt *et al*. [27] reported that an increase in procalcitonin levels on day 1, compared with baseline, had a sensitivity of 41% and a specificity of 85% for diagnosing VAP, with positive and negative predictive values of 68% and 65%, respectively. Due to differences in study design and patient selection, direct comparison of results is limited, but both studies indicate that PCT has significant value in the early prediction of VAP.

Sputum culture is considered the gold standard for the clinical diagnosis of bacterial pneumonia, but it is relatively costly and requires at least two days to obtain results, which is not conducive to early clinical intervention. Compared with sputum culture, sputum smear examination is more economical and rapid, requiring only about ten minutes to obtain test results. It can provide earlier etiological data and facilitate the early diagnosis of pneumonia types. However, the quality and quantity of information yielded by Gram-stained smears depend on the experience and expertise of the personnel conducting the tests, which introduces a risk of misdiagnosis. Cao *et al*. [28] analyzed 224 sputum samples from 125 children with lower respiratory tract infections using Gram-stained smears and cultures, reporting a sensitivity and specificity of 85.5% and 87.2%, respectively, for the Gram-stained sputum smear method. In our study, the sensitivity of sputum smears was 80.00%, the specificity was 77.90%, and the AUC was 0.745. The differences between our results and those of Cao *et al*. [28] may be related to sputum collection methods and sample size differences.

To the best of our knowledge, this is the first study to combine the NLR, PCT, CRP, and sputum smear results to predict VAP after Stanford type A aortic dissection surgery. The results suggest that the combined application of these markers has high sensitivity (93.33%) and specificity (87.36%) in predicting the occurrence of VAP. The combined prediction using the NLR, PCT, CRP, and sputum smear is significantly better than using each indicator alone, indicating that this approach is highly valuable for the early diagnosis of VAP after Stanford type A aortic dissection surgery.

The results of this study revealed that *gram-negative bacteria*, mainly *Acinetobacter baumannii*, *Klebsiella pneumoniae*, and *Stenotrophomonas maltophilia*, were the main infectious pathogens of VAP. *Acinetobacter baumannii* is a non-fermentative, opportunistic pathogen that is part of the normal flora of the human body and widely exists in natural and hospital environments. It has a strong ability for clonal transmission and is an important pathogen in VAP [29, 30]. *Stenotrophomonas maltophilia *is an emerging pathogen listed as a public health concern, infecting critically ill patients and often showing resistance to antimicrobial treatments [31]. *Klebsiella pneumoniae* is a gram-negative bacterium that often colonizes the respiratory, urinary, and intestinal tract. It is an opportunistic pathogen that usually affects immunosuppressed patients and is a common cause of hospital-acquired infections, including VAP [32, 33]. In recent years, due to the increasingly serious misuse of antibacterial drugs and other issues, the difficulty of clinical antibacterial treatment has increased. Therefore, targeted treatment should be implemented based on the distribution characteristics of pathogenic bacteria and drug resistance patterns in the hospital.

The limitations of this study are as follows: owing to the relatively low incidence of acute type A aortic dissection and the single-centre design, the sample size was relatively small and may be subject to selection bias. Multicentre studies with larger samples are needed for further confirmation. Additionally, as this study was retrospective, there is a risk of recall bias, which may affect the reliability and accuracy of the results. In this study, although the combination of multiple inflammatory markers proved more effective than the use of single markers, the clinical value of this approach is still limited. For future research, we consider expanding the sample size and adopting advanced methods to build more robust risk assessment models.

## 5. Conclusions

The study demonstrates that monitoring CRP, PCT, NLR, and sputum smear results is an effective strategy for the early prediction of VAP in patients post-Stanford type A aortic dissection surgery. The combined diagnostic approach improves accuracy, enabling early and appropriate clinical interventions. Recognizing that *gram-negative bacteria* are the main causative agents of VAP informs the selection of targeted antibiotics, which is vital for effective treatment and combating antimicrobial resistance. These findings contribute to better clinical management of VAP and emphasize the importance of ongoing surveillance of infection markers and pathogen profiles in critically ill patients. Future research should focus on validating these results in more extensive multicenter studies and exploring additional biomarkers or methods to improved early detection and treatment of VAP.

## Availability of Data and Materials

The original contributions presented in the study are included in the article/supplementary material, further inquiries can be directed to the corresponding author.
